# A Mutation in *cnot8*, Component of the Ccr4-Not Complex Regulating Transcript Stability, Affects Expression Levels of Developmental Regulators and Reveals a Role of Fgf3 in Development of Caudal Hypothalamic Dopaminergic Neurons

**DOI:** 10.1371/journal.pone.0113829

**Published:** 2014-12-05

**Authors:** Peter Koch, Heiko B. Löhr, Wolfgang Driever

**Affiliations:** 1 Developmental Biology, Institute Biology I, Faculty of Biology University of Freiburg, Hauptstrasse 1, D-79104 Freiburg, Germany; 2 BIOSS–Centre for Biological Signaling Studies, University of Freiburg, Schänzlestrasse 18, D-79104 Freiburg, Germany; University of Birmingham, United Kingdom

## Abstract

While regulation of the activity of developmental control genes at the transcriptional level as well as by specific miRNA-based degradation are intensively studied, little is known whether general cellular mechanisms controlling mRNA decay may contribute to differential stability of mRNAs of developmental control genes. Here, we investigate whether a mutation in the deadenylation dependent mRNA decay pathway may reveal differential effects on developmental mechanisms, using dopaminergic differentiation in the zebrafish brain as model system. In a zebrafish genetic screen aimed at identifying genes controlling dopaminergic neuron development we isolated the *m1061* mutation that selectively caused increased dopaminergic differentiation in the caudal hypothalamus, while other dopaminergic groups were not affected. Positional cloning revealed that *m1061* causes a premature stop codon in the *cnot8* open reading frame. Cnot8 is a component of the Ccr4-Not complex and displays deadenylase activity, which is required for removal of the poly (A) tail in bulk mRNA turnover. Analyses of expression of developmental regulators indicate that loss of Cnot8 activity results in increased mRNA *in situ* hybridization signal levels for a subset of developmental control genes. We show that in the area of caudal hypothalamic dopaminergic differentiation, mRNA levels for several components of the FGF signaling pathway, including Fgf3, FGF receptors, and FGF target genes, are increased. Pharmacological inhibition of FGF signaling or a mutation in the *fgf3* gene can compensate the gain of caudal hypothalamic dopaminergic neurons in *cnot8^m1061^* mutants, indicating a role for Fgf3 in control of development of this dopaminergic population. The *cnot8^m1061^* mutant phenotype provides an *in vivo* system to study roles of the Cnot8 deadenylase component of the mRNA decay pathway in vertebrate development. Our data indicate that attenuation of Cnot8 activity differentially affects mRNA levels of developmental control genes.

## Introduction

The regulation of mRNA stability is one of the mechanisms for major regulatory transitions during embryonic development, clearing mRNAs characteristic for an early phase of development and facilitating the control of the next developmental phase or differentiation state by newly transcribed mRNAs [1]–[4]. *in vivo* mRNA metabolism is determined by quantity and time period of transcription, but also by mRNA turnover mechanisms including deadenylation and decapping, defining the half life of an mRNA species during which functional protein can be generated. mRNA turnover is a highly sophisticated and carefully regulated mechanism evolved to establish and maintain the amount of functional protein required by a cell [5]. Recent interest has much focused on the roles of non-coding RNAs in controlling both mRNA translation and mRNA decay in embryogenesis [6], [7]. Whether and how enzymatic mRNA decay control mechanism [8] contribute to specific patterning or differentiation decisions during vertebrate embryogenesis is less well understood.

Deadenylation is thought to be the initial step in bulk mRNA turnover [9]–[11] and is first mediated by the Pab1p-dependent poly (A) nuclease (PAN2-PAN3) complex trimming the poly (A) tail to a length of 60 to 80 nucleotides [12], [13]. Subsequently the Ccr4-Not complex removes the remaining poly (A) tail finally exposing the mRNA to decapping and decay mechanisms. The Ccr4-Not complex is conserved from yeast to human [14]–[17]. In yeast the Ccr4-Not complex is the main deadenylase and comprises 9 core components [18]. Ccr4 associated factor 1 (Caf1) and Carbon catabolite repression factor 4 (Ccr4) are the only subunits of the Ccr4-Not complex involved in 3′ to 5′ deadenylase activity [19], [20]. Caf1 has a second function in associating Ccr4 to the Ccr4-Not complex [21]. *cnot8* and *cnot7* are homologs of the yeast *Caf1* gene in zebrafish, mouse, and human. Instead, in *Drosophila* only one homolog (*POP2*) has been identified [15], [17], [19]. In vertebrates the Ccr4-Not complex contains the three proteins Ccr4, Cnot8 and Cnot7, dispatching deadenylase function (reviewed in [11]). Whereas the biochemical roles of *cnot8* and *cnot7* in the mRNA decay pathway are well studied, and functions have been determined in cell culture (for example, [22]) as well as invertebrate systems, it so far is not well understood whether they may also contribute to differential control of mRNA turnover during development and differentiation in vertebrates. Cnot7 has been shown to be required for normal spermatogenesis in mice [23], but for Cnot8 mouse phenotypes have not been reported.

Dopaminergic (DA) neurons are intensively studied both because of the medical relevance of DA neurons for many neurological diseases, and because DA neurons are an excellent paradigm for differentiation of neurons of one transmitter phenotype in diverse regions of the brain [24]–[26]. Zebrafish have become popular as animal model to study DA differentiation, because of ease of genetic and experimental access and excellent visualization of neuronal types in the transparent embryos and larvae [27]–[34]. Most zebrafish DA groups topologically correspond to those typically found in other vertebrates. Like in mammals, DA neurons develop in the olfactory bulb (OB; mammalian A16) and in the retina (mammalian A17) of zebrafish. The prethalamic group (numbered DC1 in larvae) is homologous to mammalian neurons of the prethalamic zona incerta (A13). All DA clusters located in the posterior tuberculum in zebrafish (groups DC 2, 4, 5, and 6) require the activity of the transcription factor Orthopedia (Otp) and are homologous to the OTP-depending A11 DA in the mammalian brain [35]. The DA groups of the preoptic region and the hypothalamus (groups DC 3 and 7) in zebrafish correlate with mammalian A12 and A14 DA groups. Zebrafish however lack ventral midbrain DA neurons homologous to the mammalian A8-10 group. Instead, an additional group of DA neurons exists in the zebrafish striatum [27], [29], [36]. Most studies in zebrafish have focused on transcriptional control of DA groups [29], especially of the A11-type DA neurons [37]–[42], and on signaling mechanisms [43],[44]. Studies on signaling mechanisms have provided insight into mechanisms controlling the number of DA neurons for the A11-type DA group [42], [44], [45]. In contrast, very little is known about mechanisms that control DA neuron number in other anatomical groups, including the hypothalamic ones.

In a zebrafish genetic screen for mutations affecting expression pattern and level of *tyrosine hydroxylase (th)* mRNA as marker for DA and noradrenergic (NA) neurons, we identified a mutation in the *cnot8* gene. The mutant phenotype is attenuated during early developmental stages by maternally derived functional Cnot8 protein, which gradually decays as development proceeds. *cnot8^m1061^* mutant embryos display increased *th* transcript levels and increased numbers of DA neurons particularly in the caudal hypothalamus. The hypothalamic DA phenotype may be caused by direct effects on *th* mRNA stability, or by changes in levels of developmental regulators controlling formation of caudal hypothalamic dopaminergic neurons. We find that *fgf3* as well as FGF receptor genes are expressed at higher levels in the caudal hypothalamus in *cnot8^m1061^* mutant embryos. Our data suggest that Cnot8, as Caf1 in yeast [20] and POP2 in *Drosophila* [17], may have a function in mRNA turnover in zebrafish. Rendering the Cnot8 protein non-functional may result in decreased mRNA decay rates for many but not all developmental regulators, and thus accumulation of those mRNAs. The detailed analysis of genes affected by Cnot8 deficiency in the caudal hypothalamus led us to identify FGF signaling, and specifically Fgf3, as pathway contributing to the specification of the proper number of DA neurons in the caudal hypothalamus.

## Results

### The zebrafish mutation *m1061* causes increased catecholaminergic differentiation marker transcript levels

**Figure 1:**
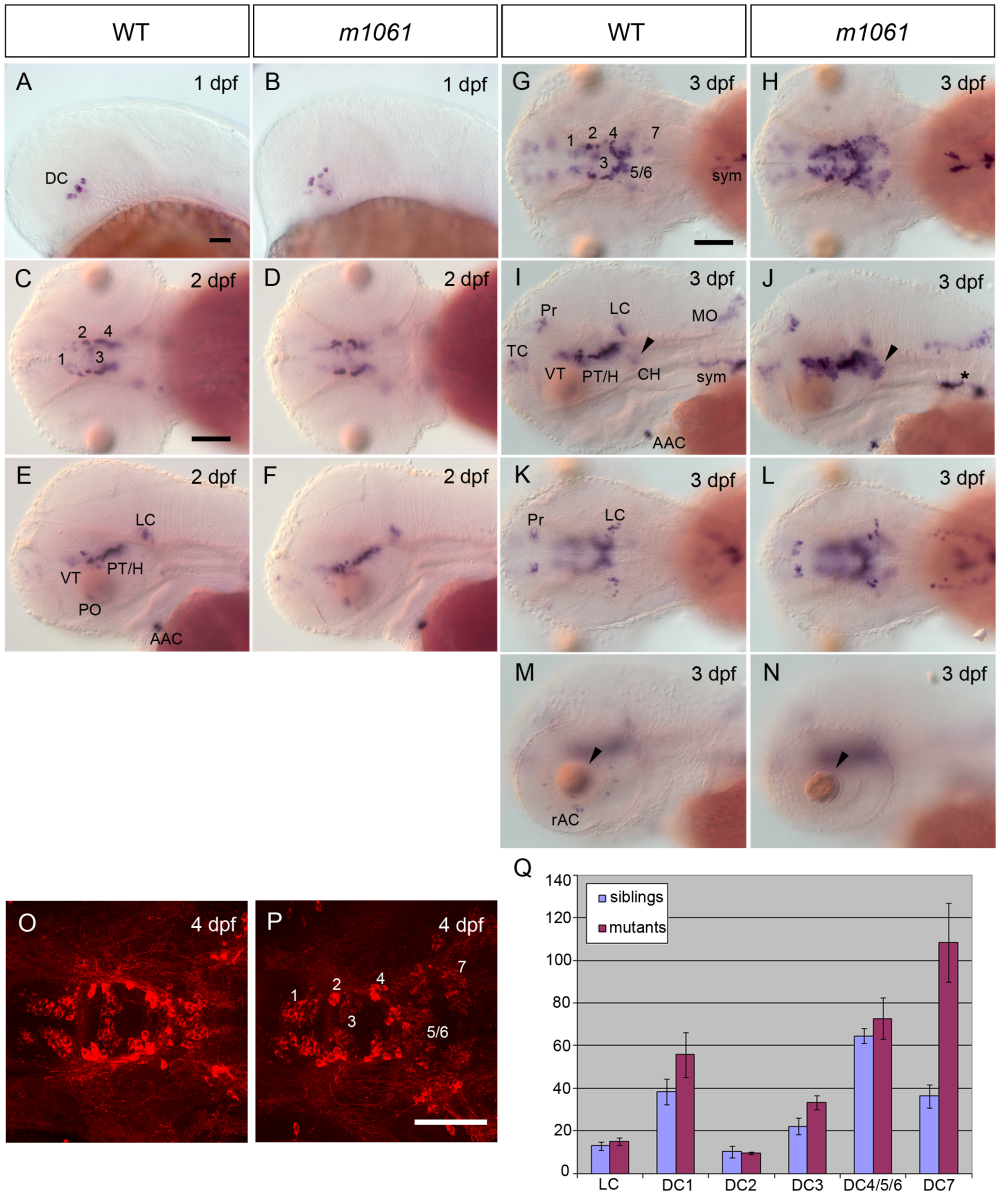
*m1061* mutants show increased *th* expression and increase in number of hypothalamic DA neurons from 3 dpf onwards. Analysis of *th* gene expression in *cnot8^m1061^* mutants and WT siblings as indicated in header. (A–F) *th* transcript levels are not altered in *m1061/m1061* mutants at 1 and 2 dpf. Embryos were genotyped by PCR. (G–N) *m1061* mutants at 3 dpf display stronger WISH signal indicating increased *th* mRNA levels in DA groups 1 to 7 (arrowhead) and NA sympathetic cells (asterisk). (K, L) NA locus coeruleus (LC) and DA pretectal cells (Pre) do not display altered *th* expression levels in *m1061* mutant embryos. (M, N) The number of DA retinal amacrine cells (rAC) is reduced in *m1061* mutant embryos. Additionally *m1061* mutants display a lens defect (arrowheads). Genotypes were inferred by *th* WISH analysis. (O, P) anti-TH immunohistochemistry of *m1061* mutant embryo and WT sibling at 4 dpf document DA neurons in the ventral diencephalon. Data confirm a higher cell number of DC7 DA neurons in the caudal hypothalamus of *m1061* mutants. Dorsal view z-projections of partial confocal stacks representing the ventral diencephalon are shown. (A, B, E, F, G, H, M, N) lateral views, anterior at left; (C, D, G, H, K.L, O, P) dorsal views, anterior at left. Scale bars 100 µm in A for A, B; in C for C–F; in G for G–N; in P for O, P. Abbreviations: DC - early diencephalic DA group (DC2, DC4), 1 - ventral thalamic DA group, 2,4,5,6 posterior tubercular and hypothalamic Orthopedia-dependent DA groups, 3 - medial hypothalamic DA group, 7 - caudal hypothalamic DA group, VT - ventral thalamus, PT/H - posterior tuberculum/hypothalamus, PO - preoptic area, AAC - arch associated catecholaminergic neurons/carotid body, sym - sympathetic NA neurons, Pr - pretectum, TC - telencephalon, CH - caudal hypothalamus, LC locus coeruleus, MO - medulla oblongata, rAC - retinal amacrine cells. (Q) Quantification of CA cell numbers in *m1061* embryos at 4 dpf. Cell counts of DC1-7 and LC TH-expressing cells. Bars show the average number of CA neurons in five *m1061* mutants and five WT sibling embryos. Error bars indicate standard deviation. Significance was evaluated using Mann-Whitney test (see text and Table S1).

**Figure 2:**
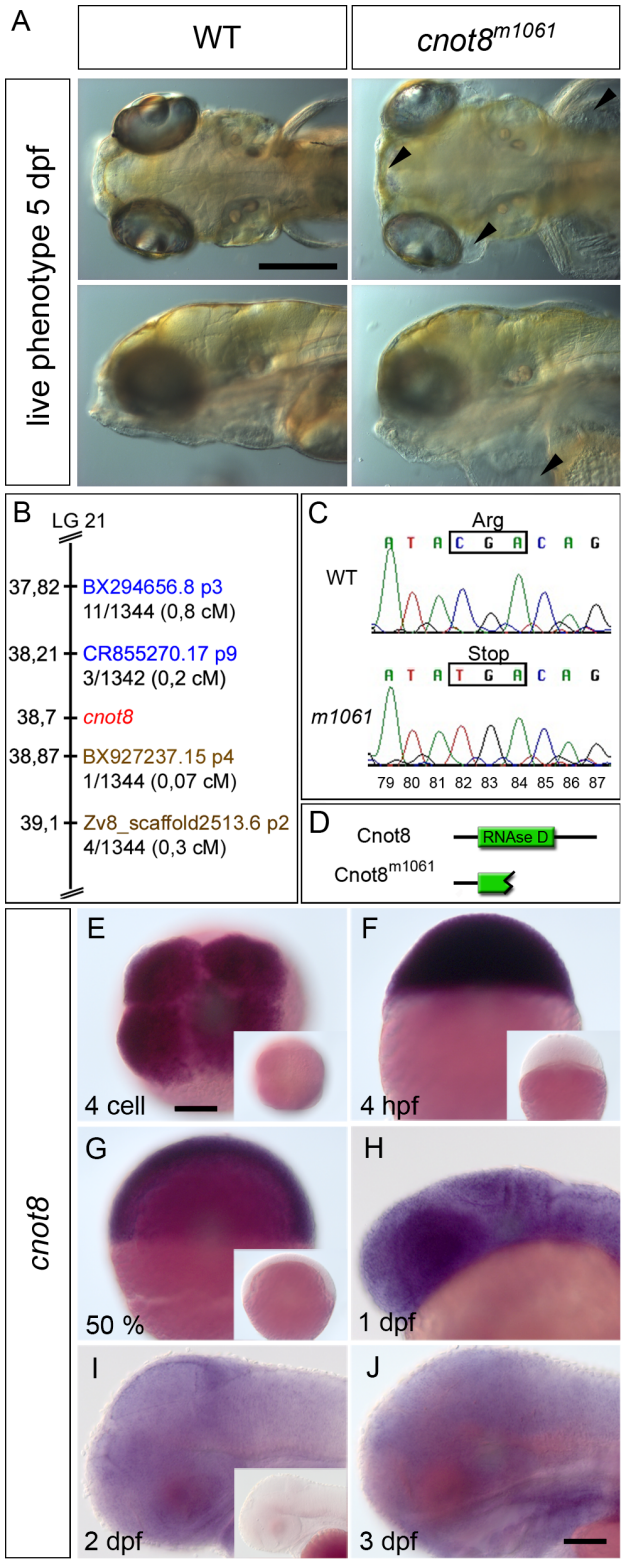
The live phenotype and positional cloning of the *cnot8^m1061^* mutation. (A) Comparison of *cnot8^m1061^* mutant to wild-type sibling embryos at 5 dpf; *cnot8^m1061^* mutants display edema formation in the eyes and brain (top row, arrowheads) as well as cardiac and yolk sac (bottom row, arrowhead). Top row dorsal view, bottom row lateral view, anterior to the left. Scale bar 100 µm. (B) Scheme of the *m1061* genetic interval. Gene and marker positions were obtained from Ensembl Zv8. CR855270.17 p9 and BX927237 p4 define the interval which comprises the genes *zgc:77151*, *MRP L22*, *gemin5*, *cnot8*, *kibra/WWC1*, *ARL10* and *Nop16* (data not shown). Proximal SSLP markers are highlighted in blue. Distal SSLP markers are highlighted in brown. Numbers provided with SSLP markers reflect recombination events identified per number of meioses analyzed. (C) Sequencing of genomic DNA amplified by PCR from individual *m1061* homozygous WT and mutant embryos. The C-to-T mutation at bp 82 of the ORF results in the formation of a premature stop codon in *m1061* mutant *cnot8* ORF. Numbers (79–87) indicate ORF bp position. (D) Zebrafish Cnot8 comprises 286 amino acids and contains an RNAse D domain which is truncated in *cnot8^m1061^* mutants. (E–J) Expression analysis by WISH using *cnot8* antisense probes. Sense controls processed exactly in parallel with the antisense reactions are shown as insets in E, F, G and H to evaluate background stain intensities. (E) Maternal mRNA is detected at 4 cell stage (dorsal view). (F) Ubiquitous expression of *cnot8* mRNA at 4 hpf (lateral view). (G, H, I) *cnot8* continues to be expressed ubiquitously at 50% epiboly, 1, 2 and 3 dpf (lateral views). Scale bars 100 µm.

During a systematic genetic screen for mutations affecting development of catecholaminergic (CA) neurons in zebrafish, we identified the *m1061* mutation, which was unique in that it was the only mutation isolated which appeared to cause enhanced differentiation of selected CA neuronal groups. In the screen, which was based on detecting *tyrosine hydroxylase* mRNA expression as marker for CA neurons, the *m1061* mutation gave rise to a stronger and expanded whole mount in situ hybridization (WISH) signal for *th* expression, likely reflecting higher *th* mRNA levels and potentially also an elevated number of DA and NA neurons. At 1 and 2 days post fertilization (dpf), (Figure 1A–F) *m1061* mutants display a *th* expression pattern indistinguishable from wild-type siblings. At 3 dpf, a stronger *in situ* hybridization signal for *th* expression was observed in the diencephalic DA cell clusters of *m1061* mutants. This phenotype was especially pronounced in the caudal hypothalamic diencephalic dopaminergic cluster 7 (DC7; Figure 1G–J, arrowhead in I, J). Most other DA groups in the forebrain developed normally up to 4 dpf, with the exception of the pretectal cluster, which showed a minor increase in stain intensity (Figure 1K, L), and the retinal amacrine DA neurons, which appear to be severely reduced in *m1061* mutant embryos (Figure 1M, N). We also analyzed NA neuronal clusters, which revealed that the *th* signal for medulla oblongata (MO) and sympathetic NA neurons appeared to be stronger in *m1061* mutant embryos (Figure 1I and asterisk in J). The NA neurons of the locus coeruleus (LC) did not appear to be affected in *m1061* mutants. Whole mount immunofluorescence detection of TH was performed and confocal stacks representing the whole ventral diencephalon and rostral hindbrain were analyzed for cell numbers of diencephalic DA cell clusters and the NA cells of the LC at 4 dpf (Figure 1O–Q). As DC 4, 5 and 6 are difficult to distinguish due to close anatomical location, neurons of these groups were combined for analysis. The cell counts were analyzed for statistical significant differences in mutant embryos using the Mann-Whitney test. In *m1061* mutant embryos, a strong increase of DA cell numbers was only found for the caudal hypothalamic DC7 DA group (p = 0.008). DA neurons of DC7 showed a more than 2-fold increase in number in *m1061* mutants when compared to wild-type siblings (Figure 1Q). Milder increases in cell number were found in DA DC1 (p = 0.032) and 3 (p = 0.008; Figure 2C). The cell numbers in other DA cell clusters like DC2, 4, 5, 6 and the LC were not significantly different between *m1061* embryos and controls.

Given the pronounced changes in CA differentiation, we determined whether living *m1061* mutant larvae also develop morphological abnormalities. On the first and second day of development, the overall live morphology of *m1061* mutant embryos appear indistinguishable from wild-type siblings (data not shown). From 3 dpf on, *m1061* mutant embryos have smaller heads with smaller brain and eyes, as well as impaired branchial arch development. In 5 dpf old *m1061* mutant larvae, the eyes are significantly smaller than in wild-type siblings, and edema formation was observed in several tissues including the eyes, brain, heart sack, yolk sack and the intestine (Figure 2A). In addition, *m1061* mutants have defective swim bladder formation and reduced heart beat frequency. Further, *m1061* mutant larvae from 4 dpf on have a reduced motility in comparison to wild-type sibling larvae (data not shown). Mutant larvae die soon after 5 dpf and cannot be raised to feeding larvae.

### The *m1061* mutation affects the *cnot8* genetic locus

To identify the gene affected by the *m1061* mutation, we mapped it using bulked segregant analysis and Simple Sequence Length Polymorphism (SSLP) genetic markers [46], and identified linkage to chromosome 21. Fine mapping of the *m1061* mutation required the generation of additional SSLP markers based on published sequence (Materials and Methods). The *m1061* critical genetic interval was defined by the proximal marker CR855270.17 p9 (genetic distance of 0.2 cM) and the distal marker BX927237 p4 (genetic distance of 0.07 cM) (Figure 2B). At the approximate position of 38.7 Mb of the linkage group 21 (assembly Zv8; www.ensembl.org), we identified *cnot8* and other adjacent genes as candidate genes for the *m1061* mutation. The sequencing of cDNA from homozygous mutant *m1061* larvae identified a base change at bp 82 within the *cnot8* ORF, which resulted in changing an arginine codon to a stop codon, causing premature termination of the peptide after 27 amino acids. This result was confirmed by amplification and sequencing of the corresponding genomic DNA from *m1061* mutant and wild-type sibling larvae (Figure 2C). The zebrafish Cnot8 protein consists of 286 amino acids and has one predicted functional domain which exerts exonuclease activity and comprises amino acids 13 to 240 (Figure 2D). The premature stop codon causes the formation of a truncated Cnot8 peptide which lacks most of its RNAse domain and should lack its poly (A) deadenylation function. Thus, the *m1061* allele is expected to represent a complete loss of function or null allele of *cnot8*.

*cnot8* has been reported to be broadly expressed (www.ZFIN.org ZDB-IMAGE-050809-54; [47]). To confirm whether *cnot8* is globally expressed and not in a tissue specific manner, we analyzed its expression throughout embryonic and early larval stages. *cnot8* mRNA was already detected at 4-cell stage (Figure 2E). Since transcription from the zebrafish zygotic genome starts only at midblastula transition (MBT), shortly after 3 hpf [48], this reveals maternally transcribed *cnot8* mRNA deposited into the oocyte. Therefore functional maternal mRNA derived Cnot8 protein is likely to enable proper development at early gastrula stages in *cnot8^m1061^* mutant embryos. During later embryonic and early larval stages *cnot8* was ubiquitously expressed (Figure 2F–J; insets show sense control WISH).

Based on the maternal and zygotic expression, *cnot8^m1061^* mutant embryos are expected to contain about half the amount of wild-type functional maternal protein, but no functional zygotic mRNA derived Cnot8 protein. Therefore, the mutant phenotype represents the gradual loss of functional maternally derived protein during embryonic stages, as most maternal mRNAs appear to be degraded during blastula and gastrulation stages [1]. Stable maternal derived Cnot8 protein may therefore persist well through the first two to three days of development. Therefore, we investigated whether translational block of *cnot8* mRNA by antisense Morpholino oligonucleotides directed against sequence including the start Cnot8 ATG (*MOcnot8ATG*) would cause a phenotype stronger than *cnot8^m1061^*. Injection of a low amount of *MOcnot8ATG* (1 ng) lead to a more severe morphological phenotype, while 2–8 ng amounts caused early lethality (Figure S1). The morphant phenotype was not further investigated because Morpholino-induced broadly lethal phenotypes are difficult to analyze. We also did not attempt to generate maternal and zygotic mutant embryos based on mutant germline clones, because we reasoned that Cnot deficient germline might be cell-lethal. We also investigated whether microinjection of mRNA encoding the wild-type Cnot8 protein into one-cell stage embryos would rescue the *cnot8^m1061^* mutant phenotype, but did not achieve a significant rescue (data not shown). We interpret this as injected mRNA similar to maternal mRNA being degraded during early development, and not being able to contribute to a rescue of the phenotype on the third day of development and later.

### Pattern formation and global organization of the brain appear normal in *cnot8^m1061^* mutant embryos

**Figure 3:**
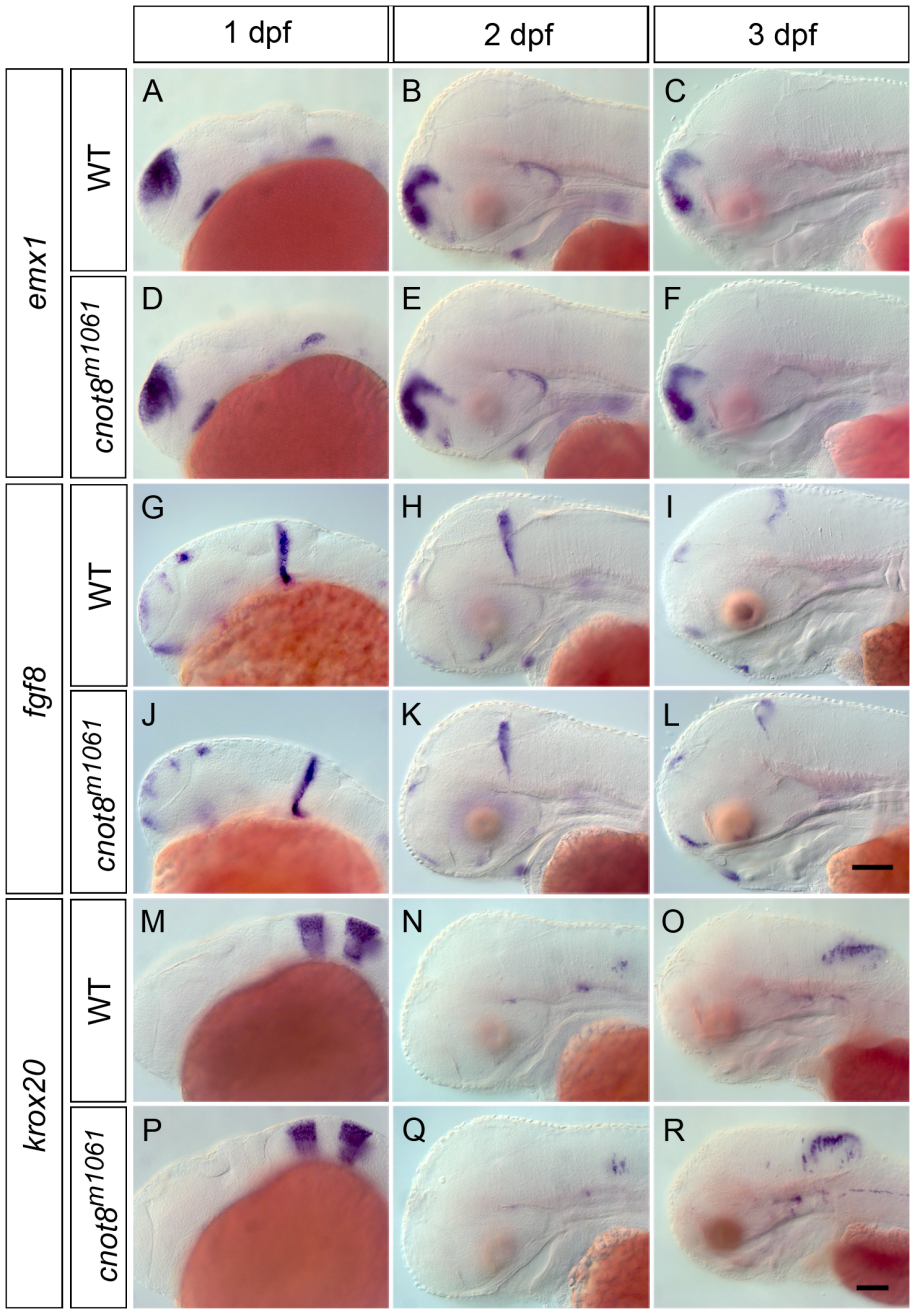
*cnot8^m1061^* mutant embryos are not generally delayed in development. Gene expression analysis of *emx1*, *fgf8* and *krox20/egr2b* in *cnot8^m1061^* mutants and wild-type siblings at 1, 2 and 3 dpf. (A–F) *emx1*, (G–L) *fgf8* and (M–R) *krox20/egr2b*. Embryos were genotyped by PCR. All pictures show lateral views. Scale bars 100 µm.

To identify whether pattern formation and morphogenesis of the brain proceed normally during the first three days of development, we investigated the expression of genes involved in pattern formation and regionalization of the brain in *cnot8^m1061^* mutant embryos. To determine whether changes in WISH signal may be significant, we measured mean intensities of WISH signal within defined anatomical domains of marker gene expression in five or more images of control and experimental (mutant) embryos, and analyzed data for significant differences in mean intensities using the Mann-Whitney test (see Methods; all measurements and statistical analyses are documented in Table S1). We used *emx1* for telencephalic development [49], *krox20/egr2b* as a marker for rhombomeres 3 and 5 [50] and *fgf8a* as a marker for prominent signaling centers like the mid-hindbrain boundary, anterior neural ridge and optic stalk [51]. In 1 and 2 dpf old *cnot8^m1061^* mutant and wild-type sibling embryos the expression pattern of these genes was indistinguishable (Figure 3). In contrast, at 3 dpf a slightly stronger WISH stain was observed for *emx1* and *krox20/egr2b* in *cnot8^m1061^* mutant embryos compared to wild-type, while *fgf8a* expression appeared not affected. Measurements revealed that the WISH signal was significantly stronger in the telencephalic *emx1* domain at 3 dpf (p = 0.032), and at 2 dpf (p = 0.017) and 3 dpf (p = 0.009) for the hindbrain *krox20/egr2b* domain.

**Figure 4:**
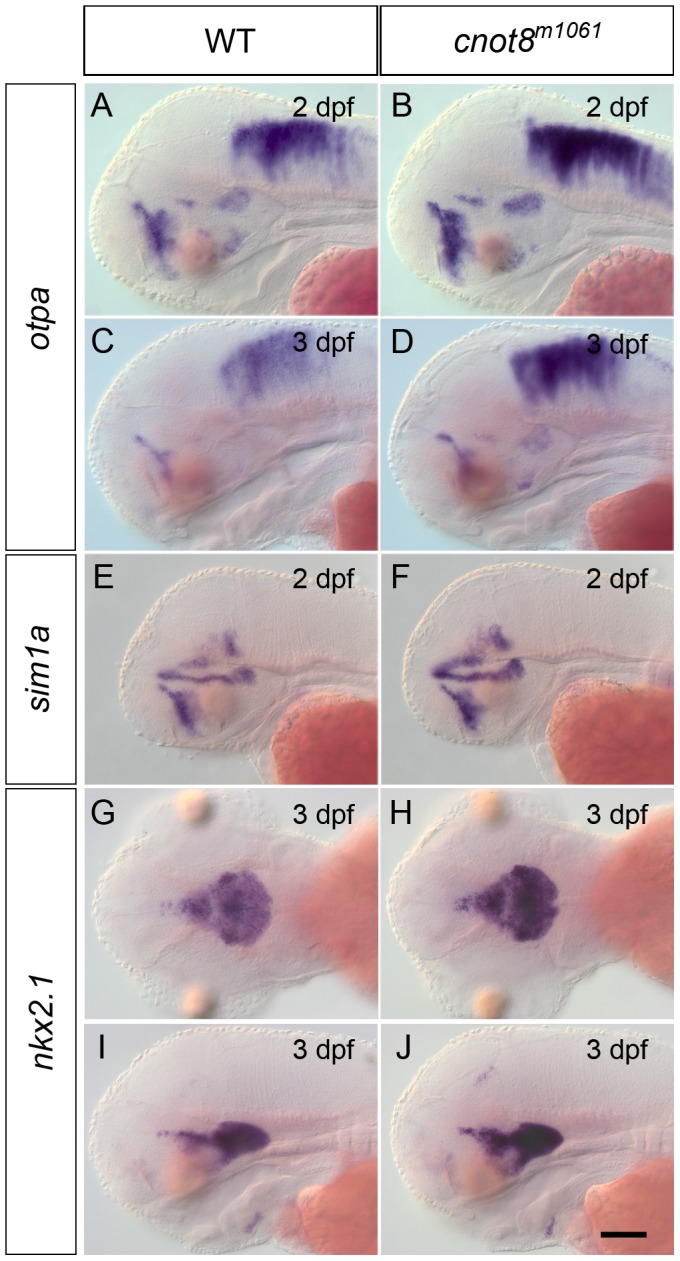
*sim1a*, *otpa and nkx2.1a* expression in *cnot8^m1061^* embryos and WT siblings. Analysis of *otpa* (A–D), *sim1a* (E, F) and *nkx2.1a* (G–J) gene expression in *cnot8^m1061^* mutant embryos and wild-type siblings, stages as indicated. (A–F, I, J) lateral views. (G, H) dorsal views. Embryos were genotyped by PCR. Scale bar 100 µm.

Given that the most severe dopaminergic phenotype was observed in the caudal hypothalamus, we analyzed expression of transcription factors involved in dopaminergic differentiation and in hypothalamic development. *otpa* and *sim1a* have been shown to encode transcription factors required for the specification and differentiation of a subset of DA neurons in the ventral diencephalon in zebrafish [41], [52]. At 2 dpf, *otpa* is expressed in several ventral diencephalic domains within the dorsal posterior tuberculum, hypothalamus, and ventral pituitary [52]. In *cnot8^m1061^* mutant embryos *otpa* is expressed in the same spatial pattern but at stronger WISH signal intensity as compared to wild-type siblings (Figure 4A, B; preoptic domain not significantly different, but hindbrain WISH signal significantly stronger, p = 0.008). At 3 dpf expression levels of *otpa* decline and the corresponding domains in the posterior tuberculum and hypothalamus are very faint in wild-type siblings, while *otpa* WISH signal in these domains is detected at higher levels in *cnot8^m1061^* mutants (Figure 4C, D; preoptic area p = 0–004). The *otpa* expression domain in the hindbrain also has a stronger WISH stain in *cnot8^m1061^* mutants embryos compared to wild-type siblings (hindbrain p = 0.002). Expression analysis of *sim1a* at 2 dpf also revealed a slightly stronger staining in *cnot8^m1061^* mutant embryos in comparison to wild-type siblings, while the expression pattern was normal (Figure 4E, F; posterior tuberculum p = 0.004). We further analyzed *nkx2.1a* expression as a marker for the hypothalamus [53]. The analysis revealed that at 3 dpf the hypothalamus in *cnot8^m1061^* mutant embryos and wild-type siblings are of equal size (Figure 4G–H). However, we observed a slightly stronger *nkx2.1a* WISH signal in *cnot8^m1061^* mutant embryos as compared to wild-type siblings (hypothalamus p = 0.008). In summary, it appears that patterning and regionalization of the brain occur normally in *cnot8^m1061^* mutant embryos, while, as judged from WISH stain intensities, the transcript levels of some genes, including the transcription factors *sim1a, otpa*, and *nkx2.1a*, appear to be increased.

### Selected neuronal cell types are affected in *cnot8^m1061^* mutant embryos

We analyzed whether also other neuronal cell types in addition to DA cells were affected in *cnot8^m1061^* mutants. We focused on neurons in the hypothalamus with regulatory links to dopaminergic specification. The transcription factors Sim1 and Otp have been shown to be required for the development of dopamine, CRH, and Oxytocin secreting neurons in mammals [52], [54]–[56] and zebrafish [41], [52], [57], [58]. In addition we analyzed serotonergic neurons for comparison.

**Figure 5:**
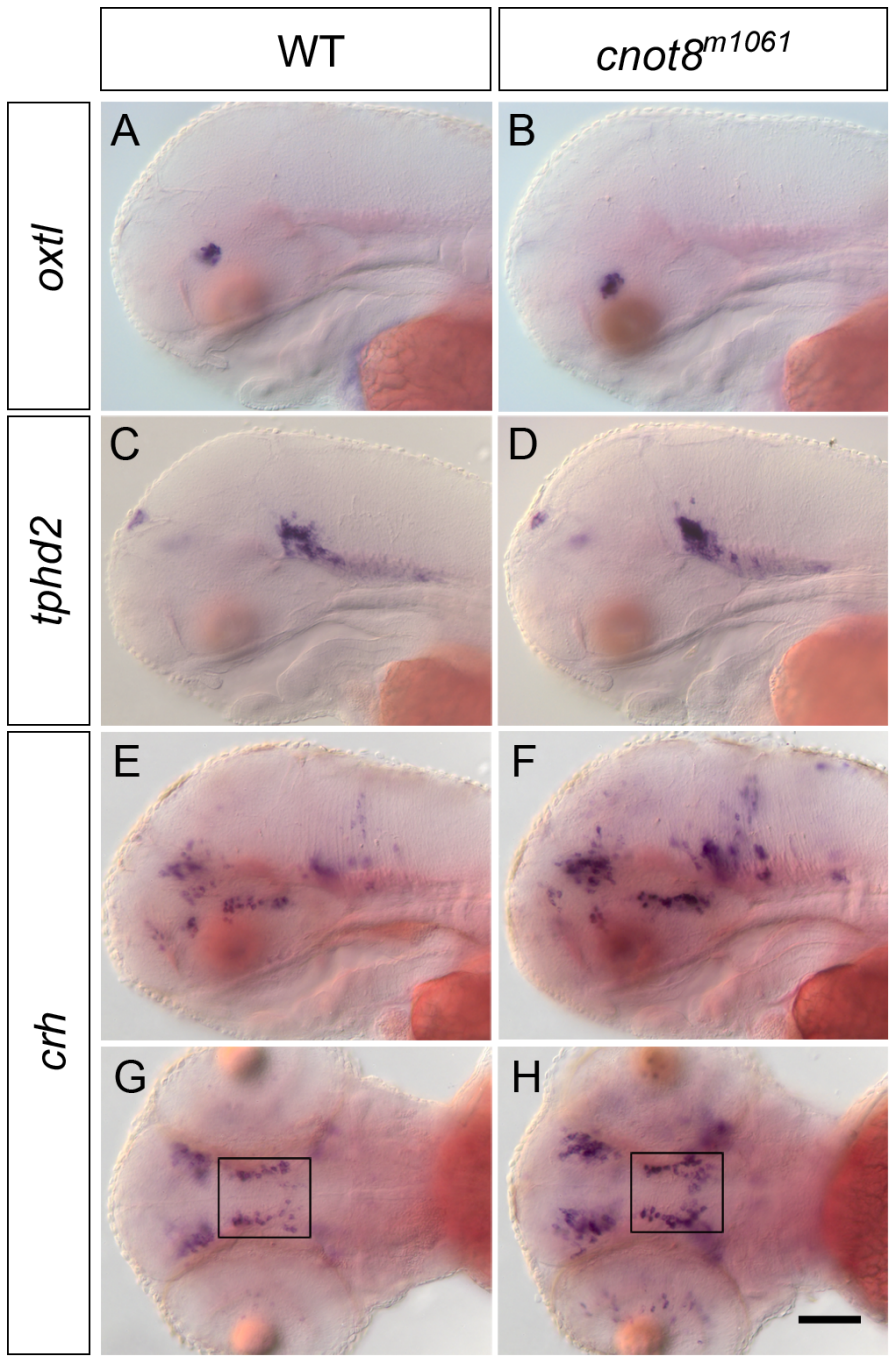
*oxtl*, *tphd2* and *crh* gene expression in *cnot8^m1061^* embryos at 3 dpf. (A, B) *oxtl*, (C, D) *tphd2* and (E–H) *crh* WISH expression analysis in wild-type siblings and *cnot8^m1061^* mutants. (A–F) lateral views. (G, H) dorsal views. Boxes indicate areas of cell counts. Sibling WT embryos developed on average 34.6 *crh* neurons and *cnot8^m1061^* mutants developed on average 59.4 *crh* neurons. Significance was evaluated by Mann-Whitney test (p = 0.008). Embryos were genotyped by PCR. Scale bar 100 µm.

Oxtl/Isotocin is the homolog of oxytocin In zebrafish [59]. *oxtl* neurons have been shown to form in two distinct areas in the preoptic region of the hypothalamus [59]. The comparison of *cnot8^m1061^* mutant embryos and wild-type siblings and mutants revealed that the *oxtl* expression pattern is not altered at 3 dpf in mutant embryos, but the WISH signal was slightly darker (Figure 5A–B; p = 0.016).

*crh* expression in early zebrafish brain development has been analyzed in detail in comparison to *th* and *oxtl* gene expression [60]. At 3 dpf *crh* is expressed in the telencephalon, posterior tuberculum, hypothalamus, thalamus, epiphysis, midbrain tegmentum, rostral hindbrain and retina. We performed in situ hybridization to analyze the development of *crh* expressing neurons in *cnot8^m1061^* mutants at 3 dpf. The analysis revealed that *crh* WISH stain intensity was increased in *cnot8^m1061^* mutant embryos in all cell clusters (Figure 5E–H; p = 0.004). To address whether the stronger signal may be caused by an increase in mRNA levels or the formation of additional CRH neurons, we counted *crh* expressing cells in proximity to DA neurons in the posterior tuberculum and hypothalamus (boxes in Figure 5G, H). In this region, we identified approximately twice the number of *crh* neurons in *cnot8^m1061^* mutant embryos in comparison to wild-type siblings (Figure 5 legend; Table S1; p = 0.008).

Serotonergic neurons are characterized by Tryptophan hydroxylase expression, the rate-limiting enzyme in neurotransmitter synthesis. In zebrafish two genes encoding Tryptophan hydroxylase, *tphd1* and *tphd2*, have been identified and their expression analyzed [61], [62]. *tphd2* is a marker for serotonergic neurons in the raphe nucleus and epiphysis. The analysis of *tphd1* and *tphd2* expression in *cnot8^m1061^* mutant embryos did not reveal any significant differences in expression compared to wild-type siblings. (Figure 5C, D and data not shown).

In summary, we conclude that different neuronal cell types are differentially affected in *cnot8^m1061^* mutant embryos: While serotonergic (*tphd2*) neurons form normally, CRH and dopaminergic neurons in selected anatomical areas are enhanced in number, and *oxtl* WISH signal appears enhanced in oxytocinergic neurons.

### Increased *fgf3* and *fgf receptor 1, 2, 3* and *4* expression levels in *cnot8^m1061^* mutants

**Figure 6:**
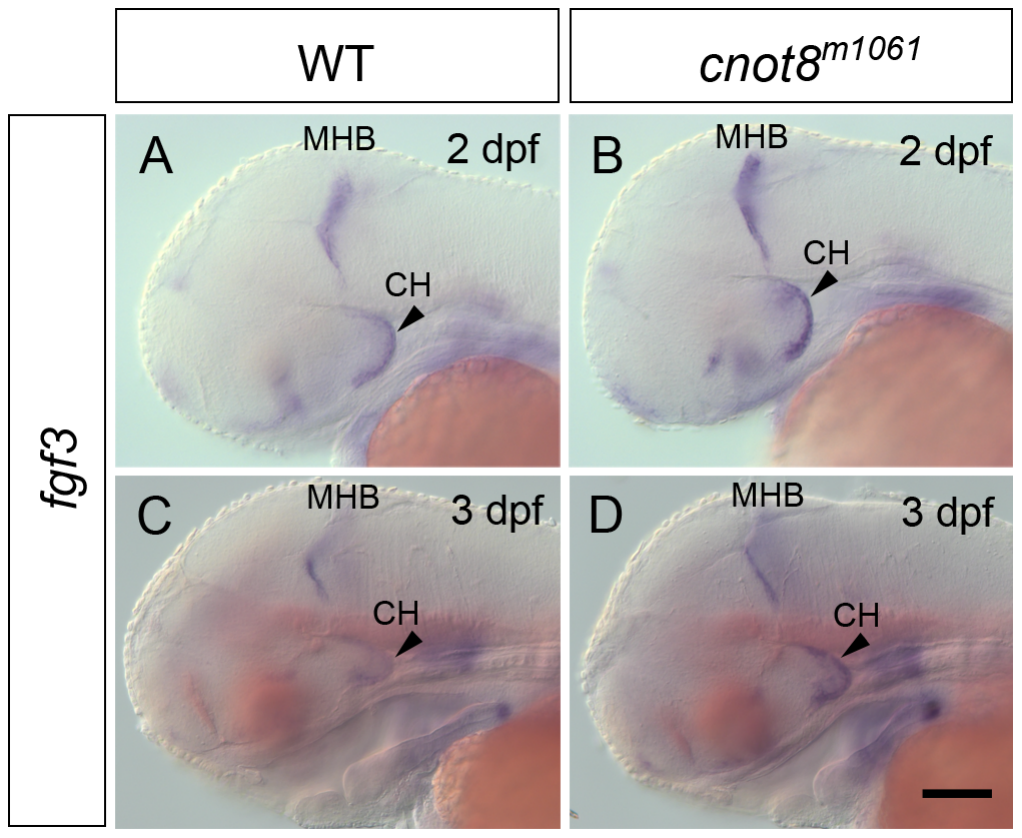
*fgf3* gene expression is increased in *cnot8^m1061^* mutants. Analysis of *fgf3* gene expression in *cnot8^m1061^* mutants and WT siblings 2 dpf (A, B), and 3 dpf (C, D). All pictures show lateral views. Embryos were genotyped by PCR. Scale bar 100 µm.

**Figure 7:**
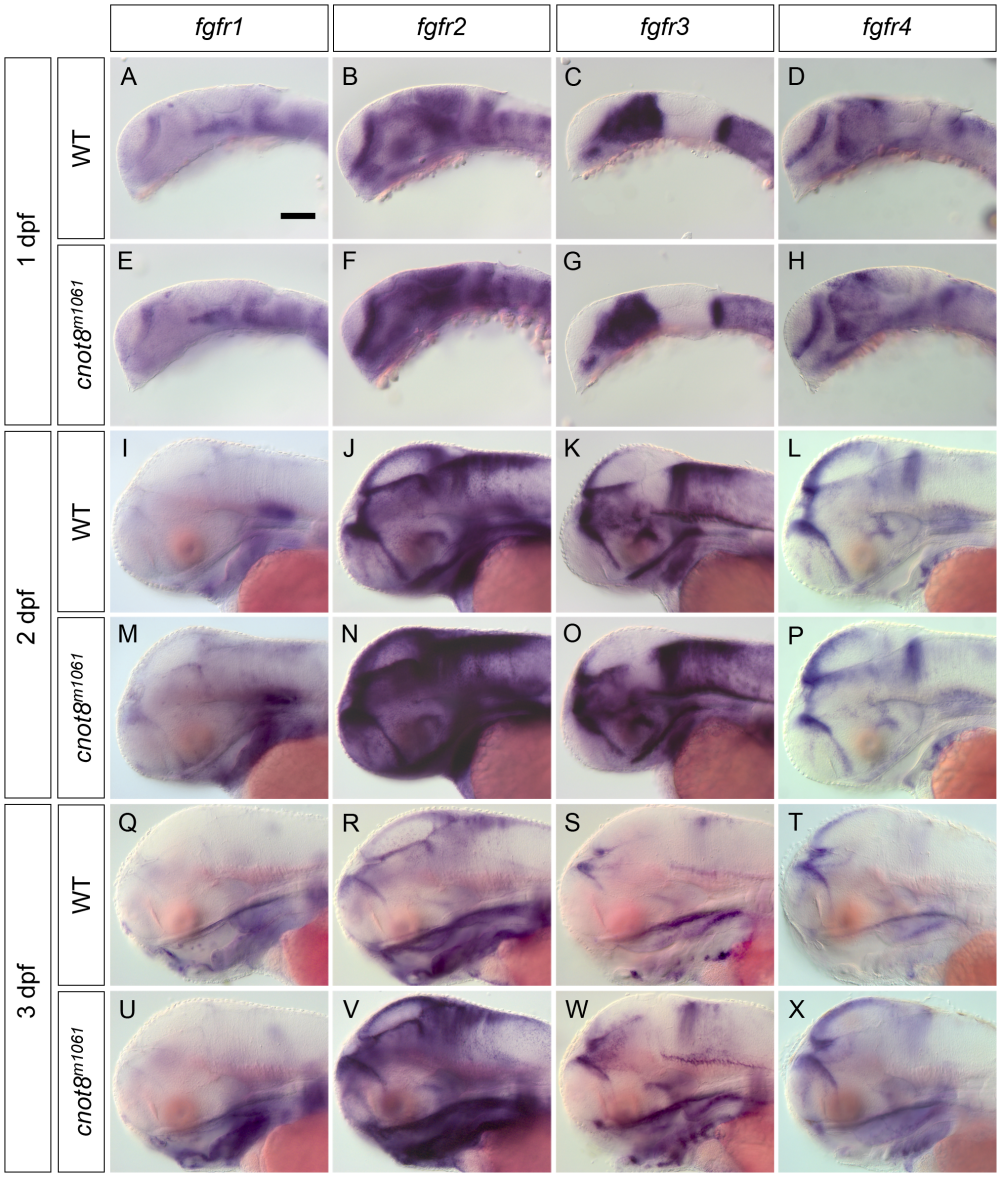
The *cnot8^m1061^* mutation affects FGF receptor expression levels. The expression of the four zebrafish FGF receptor genes *fgfr1*, *2*, *3* and *4* was analyzed by WISH in *cnot8^m1061^* mutants and WT siblings at 1, 2 and 3 dpf. All pictures show lateral views. Embryos were genotyped by PCR. Scale bar 100 µm in A for A–X.

The DA phenotype in the caudal hypothalamus was qualitatively different from other CA groups in that not just the *th* expression level was increased, but also the number of caudal hypothalamic neurons appeared increased. Therefore signaling pathways controlling DA differentiation in this anatomical region may be affected. FGF signaling namely through Fgf3 has been demonstrated to be important in caudal hypothalamus development [63]–[65]. Expression analysis of *fgf3* by WISH and quantification of signal intensity revealed that *cnot8^m1061^* mutants have a mild increase of *fgf3* WISH intensity already at 2 dpf (Figure 6A, B), both in the mid-hindbrain boundary expression domain (p = 0.008) and in the caudal hypothalamus (p = 0.008). *fgf3* WISH signal continues to be enhanced in both domains at 3 dpf (Figure 6C, D; MHB p = 0.008. cHyp p = 0.004). We also analyzed expression of FGF receptor encoding genes *fgfr1*, *2, 3* and *4* in *cnot8^m1061^* embryos. While at 1 dpf WISH signal intensities of *fgfr1*, 2, *3* and *4* are not significantly altered in *cnot8^m1061^* mutants (Figure 7A–H), at 2 dpf *fgfr1* and *fgfr2* showed increased WISH stain intensities in mutants (Figure 7I–P; *fgfr1* ventral hindbrain domain p = 0.008; *fgfr2* dorsal forebrain and midbrain p = 0.008), while *fgfr3* and *fgfr4* WISH signal levels were not altered in comparison to WT siblings. WISH signals of *fgfr1*, *2*, *3*, and *4* were up-regulated in *cnot8^m1061^* mutants at 3 dpf (Figure 7Q–X; *fgfr1* lower jaw p = 0.031; *fgfr2* dorsal forebrain/midbrain p = 0.016; *fgfr3* hindbrain p = 0.016; *fgfr4* dorsal midbrain p = 0.008).

### FGF target genes *pea3* and *erm* show elevated WISH signals in *cnot8^m1061^* mutants

**Figure 8:**
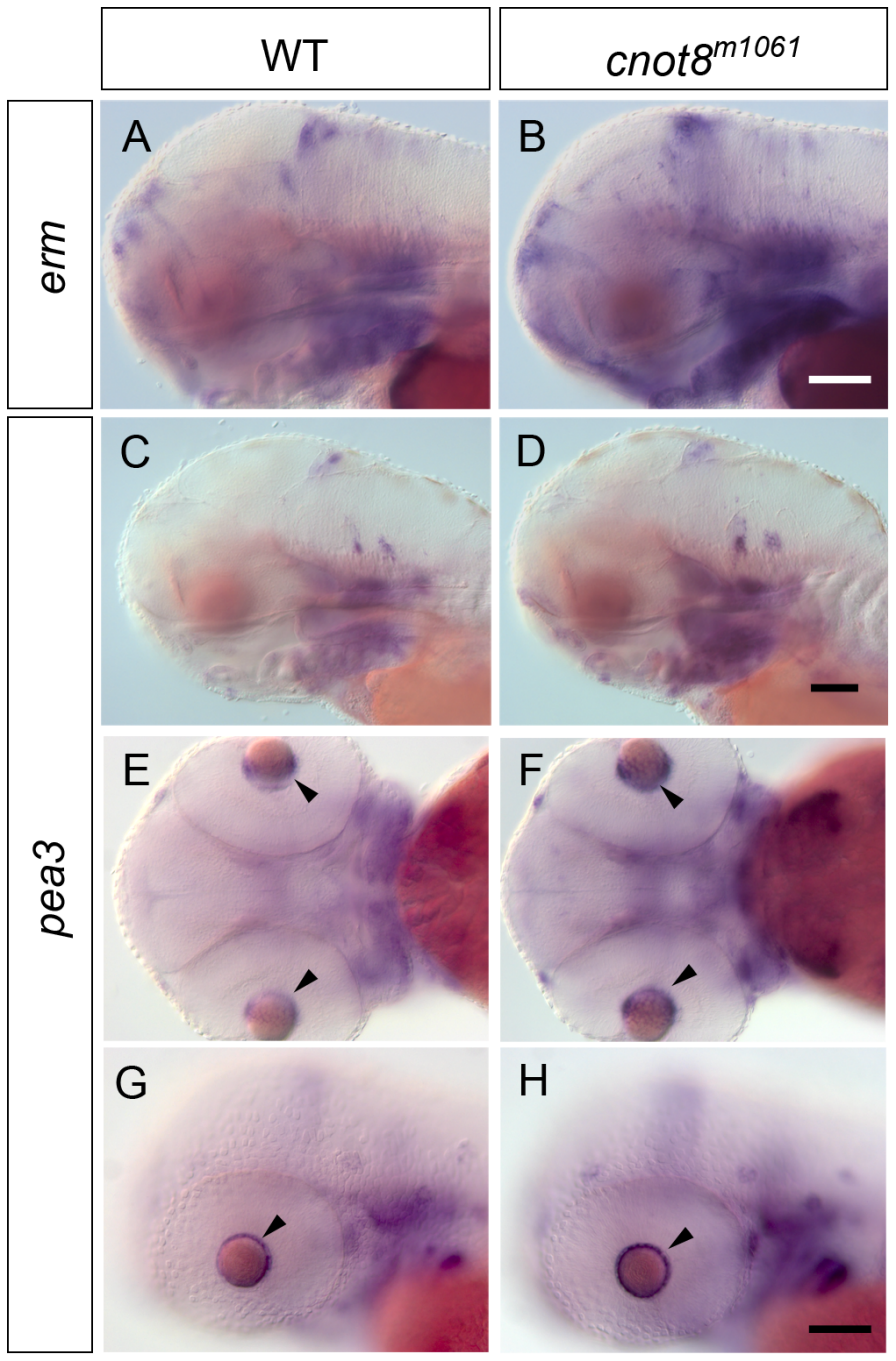
*erm* and *pea3* expression in *cnot8^m1061^* mutants and WT siblings. (A, B) *erm* expression in WT siblings and *cnot8^m1061^* mutants at 3 dpf. (C–H) *pea3* expression in WT siblings and *cnot8^m1061^* mutants at (C, D) 3 dpf and (E–H) 2 dpf. (E–G) *cnot8^m1061^* mutants show higher *pea3* mRNA levels in cells surrounding the lens in comparison to WT siblings (arrowheads). (A–D, G, H) lateral views. (E, F) dorsal views, anterior at left. Embryos were genotyped by PCR. Scale bars 100 µm in B for (A, B), in D for (C, D) and in H for (E–H).

The increase in *fgf3* as well as FGF receptor mRNA WISH signal in *cnot8^m1061^* mutants suggests that FGF signaling levels may also be increased. To address this issue we analyzed expression of the FGF downstream target genes *pea3* and *erm* by WISH in *cnot8^m1061^* embryos. Expression of ETS related protein (*erm*) was analyzed at 3 dpf (Figure 8A, B), and of polyoma enhancer activator 3 (*pea3*) at 1, 2 and 3 dpf (Figure 8C–H and data not shown). The experiments revealed that WISH signals for mRNA levels of *pea3* and *erm* were stronger in *cnot8^m1061^* mutants at 2 and 3 dpf (*erm* MHB, telencephalon, hypothalamus each p = 0.028; *pea3* cHyp p = 0.008, lens p = 0.029). *pea3* WISH signal was not increased at 1 dpf (data not shown). Increased expression of downstream targets of the FGF signaling pathway suggest increased FGF signaling levels in *cnot8^m1061^* mutants, although due to the nature of the *cnot8^m1061^* mutation in a gene affecting transcript stability, we cannot exclude that the increased *pea3* and *erm* signals may be caused by increased transcript stability.

### SU5402 treatment of *cnot8^m1061^* embryos results in reduced numbers of DC7 DA neurons but not DC1-6 DA neurons

**Figure 9:**
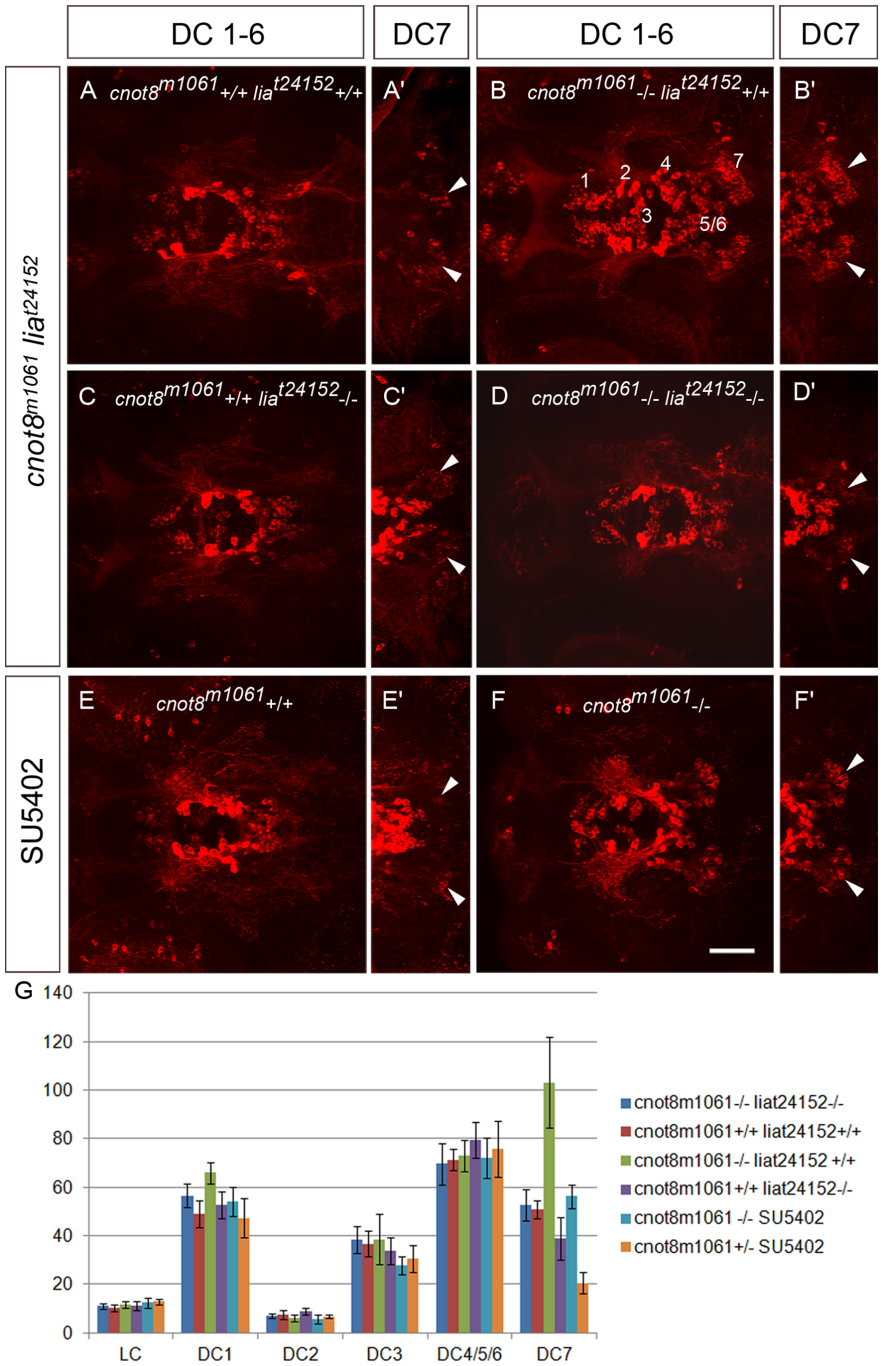
*fgf3/lia* FGF signaling mediates the increase in caudal hypothalamic DC7 DA neurons in *cnot8^m1061^* mutant embryos. (A–F) Analysis of DA neurons in *cnot8^m1061^* mutant embryos combined with a mutation in the *fgf3* locus (*lia^t24152^*) or pharmacological suppression of FGF signaling by SU5402. Embryos were fixed at 4 dpf, stained by anti TH immunofluorescence and DA neurons documented by confocal microscopy. Shown are Z-projections of confocal stacks representing the ventral diencephalic DA groups 1 to 7 (dorsal views). Scale bar 50 µm. (A–D) DA neurons in WT, *cnot8^m1061^* or *lia^t24152^* single mutant, and *cnot8^m1061^*, *lia^t24152^* double mutant embryos. Double mutant embryos show loss of *cnot8^m1061^*-mediated increase of DC7 caudal hypothalamic DA neurons. (E, F) DA neurons in WT and *cnot8^m1061^* mutant embryos treated with SU5402 from 42 to 48 hpf. Inhibition of FGF signaling by SU5402 reduces the number of DC7 caudal hypothalamic DA neurons in *cnot8^m1061^* mutants below WT levels. (G) Quantification of effects on CA neurons by cell counting of forebrain DA neuronal clusters and locus coeruleus NA neurons in genetic and experimental conditions as indicated in the index at right. Each bar shows the average number of CA neurons in five independent embryos for each experimental condition. Error bars indicate standard deviation. Average DC7 cell numbers: *cnot8+/+ lia +/+* 50.8 (WT control); *cnot8-/- lia +/+* 103.2; *cnot8-/- lia -/-* 52.6; *cnot8+/+ lia -/-* (38.8); *cnot8+/+* SU5402 20.6; *cnot8-/-*SU5402 56.2. Significance was evaluated by Mann-Whitney test. The cell count in single mutant *cnot8^m1061^* (-/-)*lia^t24152^* (+/+) is significantly different from single mutant *cnot8^m1061^* (+/+) *lia^t24152^* (-/-) embryos (p = 0.008). Comparison of *cnot8^m1061^*, *lia^t24152^* double mutant and WT embryos reveal no significant difference (p = 0.45). For SU5402 treatments, the number of DC7 DA neurons differs significantly between WT controls and SU5402 treated WT (p = 0.008) and between *cnot8^m1061^* and SU5402-treated *cnot8^m1061^* embryos (p = 0.008). For all other catecholaminergic groups, no significant differences were observed when WT was compared to single mutants, double mutants, or SU5402-treated embryos.

To determine whether enhanced FGF signaling pathway activity may contribute to formation of supernumerary DC7 DA neurons, we treated *cnot8^m1061^* embryos with SU5402, an inhibitor of FGF signaling [66]. SU5402 was applied to embryos for 6 hours from 42 to 48 hpf, the developmental period during which a significant amount of DC7 DA neurons becomes postmitotic [44]. SU5402 treatment did not result in any significant changes in cell numbers of DC1-6 (compare Figure 9A and E). *cnot8^m1061^* mutants had an average of 103.2 DC7 DA neurons, which is a 2-fold increase in DC7 cells in comparison to WT siblings (compare Figure 9A' and B'; p = 0.008). SU5402 treated *cnot8^m1061^* mutant embryos developed an average of 56.2 DC7 neurons, while SU5402 treated WT siblings contained an average of 20.6 DC7 neurons (compare Figure 9B' and F'; p = 0.008). Thus *cnot8^m1061^* mutants treated with SU5402 had only half the amount of DC7 neurons in comparison to non-treated *m1061* mutants (p = 0.008). However, *cnot8^m1061^* mutants treated with SU5402 still showed a 2.6 fold increase in cell number in comparison to SU5402 treated WT siblings (compare Figure 9E' and F'; p = 0.008). The significant reduction of DA neurons in both *cnot8^m1061^* and WT embryos by SU5402 treatment by about 50% indicates that FGF signaling may be involved in formation of DC7 DA neurons. The increase in DA neuron number in *cnot8^m1061^* may in part be mediated by enhanced FGF signaling.

### Loss of Fgf3 signaling in *cnot8^m1061^ lia(fgf3)^t24152^* double mutants reduces DC7 cell numbers almost to WT levels

To specifically investigate if Fgf3 signaling is involved in the formation of the DC7 DA *cnot8^m1061^* mutant phenotype we used the *lia* mutation, which eliminates Fgf3 activity [64], and generated *cnot8^m1061^ lia(fgf3)^t24152^* double mutants. Embryos were analyzed at 4 dpf by anti-TH immunofluorescene and confocal stacks were recorded. Using the confocal data, DA neurons were counted in embryos of WT, single mutant, and double mutant backgrounds (Figure 9G). In *lia(fgf3)^t24152^* mutant embryos DA neurons of DC1-6 develop normally (Figure 9A and C) indicating that the development of these neurons does not depend on Fgf3 signaling. DC7 neurons are about 20% reduced in number in *lia(fgf3)^t24152^* mutants in comparison to WT siblings (p = 0.024). Most interestingly *cnot8^m1061^ lia(fgf3)^t24152^* double mutants have on average of 52.6 DC7 DA neurons, and thus a significant reduction can be observed in comparison to *cnot8^m1061^* double mutants having an average of 103.4 DC7 DA neurons (p = 0.008). Loss of Fgf3 signaling in a *cnot8^m1061^* mutant background results in a reduction of DC7 DA neurons almost restoring DA cell numbers counted in WT genetic background (Figure 9G; cell numbers not significantly different; p = 0.45). These findings indicate that Fgf3 signaling, although not strictly required for differentiation of the caudal hypothalamic DA group DC7, has an important role in determining the number of these dopaminergic neurons.

## Discussion

In a forward mutagenesis screen we have identified a mutant which eliminates the activity of the zebrafish *cnot8* gene. We show that *cnot8* is expressed maternally and uniformly zygotically. The *cnot8^m1061^* zygotic mutant phenotype becomes progressively more severe as maternal Cnot8 activity declines. *In situ* expression analysis reveals that the mRNAs for a subset of neuronal differentiation markers, developmental transcription factors, and signals can be detected with elevated signal intensities in *cnot8^m1061^* mutant embryos. *th* as marker for dopaminergic neurons is enhanced in several DA neuronal groups, and the number of caudal hypothalamic DA neurons is significantly increased in *cnot8^m1061^* mutants. Analysis of the FGF signaling pathway in *cnot8^m1061^* mutants reveals that stabilization of *fgf3* mRNA and FGF receptors may control DA neuron number in the caudal hypothalamus. Using Fgf3 loss-of-function experiments, we confirm that Fgf3 contributes to control of caudal hypothalamic DA neuron number.

Cnot8 is a component of the Ccr4-Not complex which is conserved from yeast to human and considered to be a platform to regulate gene expression at different levels, including bulk mRNA degradation, protein ubiquitination, and transcription [67], [68]. While biochemical and cellular functions of the complex have been extensively characterized in cell lines and invertebrate model organisms, little is known whether Ccr4-Not or its subunits may contribute to tissue specific mRNA turnover or regulatory mechanisms in vertebrates. In mice, Cnot8 has been shown to contribute to proper spermatogenesis [23], revealing a potential for selective Cnot8 activity in development and differentiation. In this work we characterized the zebrafish *cnot8^m1061^* mutant allele which contains a premature stop codon in the ORF after amino acid 27, likely resulting in a null allele. The Cnot8 protein contains only one functional domain which is required for exonuclease activity and a truncation of the protein likely impedes deadenylation of bulk mRNA resulting in accumulation of transcripts. Proper Ccr4-Not complex function is substantial and necessary as only completely deadenylated transcripts are degraded and no longer translated. Our data suggest that Cnot8, similar to Caf1 in *Saccharomyces cerevisiae* and POP2 in *Drosophila melanogaster*, has a function in mRNA turnover in zebrafish.

Analysis of *cnot8* expression revealed that maternal *cnot8* mRNA is deposited in the embryo and *cnot8* is expressed zygotically at early zebrafish embryonic and larval stages in a ubiquitous fashion. Thus, Cnot8 may function in all cells of the embryo. cnot8 and other components of the Ccr4-Not complex were also reported to be expressed ubiquitously during all developmental stages at least until 3 dpf in zebrafish (www.zfin.org data base search).

The analysis of expression of several embryonic patterning genes as well as neural differentiation markers by *in situ* hybridization analysis revealed increased transcript levels in *cnot8^m1061^* mutant embryos for some but not all analyzed genes in embryos beginning from the second day of development. The lack of an early phenotype of *cnot8^m1061^* mutant embryos is likely caused by maternal rescue during the first day of development. At 3 dpf, in *cnot8^m1061^* mutants elevated WISH stain intensities and thus likely mRNA levels were detected for *th*, *crh*, *krox20*, *nkx2.1a, sim1*, *otpa*, *fgf3*, *pea3*, *fgf3* and FGF receptor genes, while gene expression levels of *fgf8* and *tphd2* were indistinguishable from WT siblings. These observations raised the question why only a subset of mRNA species accumulated in *cnot8^m1061^* mutants. Gene expression levels initially are defined by the rate of transcription. First, the amount of generated mRNA depends on the gene and developmental stage. Second, the half-life of each mRNA species is different as e.g. the removal of longer poly (A) requires more time [13]. Third, mRNA decay involves the function of deadenylating and decapping protein complexes (reviewed in [5]). The recruitment processes of mRNAs targeted for decay to these complexes remain poorly understood [69]. Different mRNA species may display different affinities toward protein complexes involved in mRNA decay. In vertebrates Cnot7 and Cnot8 are paralogs of yeast Caf1 [15], [16], [19]. In yeast, Ccr4 is associated to the Ccr4-Not complex via Caf1 [21], [70], [71]. Cnot7 may compensate loss of Cnot8 function. Addressing the functions of both Caf1 paralogs, Aslam et al. [22] performed siRNA mediated knockdown of Cnot8 and Cnot7 in MCF7 breast cancer cells. Subsequent microarray analysis revealed that single knock down of either component resulted in alteration in expression of few genes only, while combined knockdown caused altered expression in more than two hundred genes. Together, this argues that Cnot7 and Cnot8 may function redundantly in MCF7 cells. This notion is further supported by the finding that *Cnot7* knockout mice are viable with a spermatogenesis defect [23]. Expression analysis in mouse neural tissues also revealed differential expression of CNOT7 and CNOT8 in different tissues and downregulation of CNOT8 during differentiation [72]. Our finding of multiple changes in gene expression levels in *cnot8^m1061^* mutants combined with lethality of the mutation indicate that both paralogs, Cnot8 and Cnot7, are not entirely redundant in function during development.

We further investigated potential differential functins of Cnot8 in development using DA neural differentiation as model system. Early differentiating DA neurons of the DC2, 4, and 5 groups, and noradrenergic neurons of the locus coeruleus form during the first 36 hours of development, and appear normal in *cnot8^m1061^* mutant embryos. During these time periods sufficient functional Cnot8 may be present in *cnot8^m1061^* mutants as a result of maternally deposited *cnot8* mRNA. In contrast, cell counts of DA cells in the ventral diencephalon and hypothalamus showed that DA cell number was significantly increased in the caudal hypothalamic DC7 group in *cnot8^m1061^* mutants compared to WT siblings. An increase in the number of scored cells was also observed for CRH neurons, but not for serotonergic neurons located adjacent to DC7 DA neurons. Therefore, the effect of Cnot8 depletion is not selective for DA neurons, but also does not globally affect all neuronal types in this region and at this stage. The increase in number of counted cells may be caused by a true increase in number of DC7 DA neurons in mutant embryos, but may alternatively also be explained by increased *th* mRNA levels due to less decay, which may facilitate detection of newly specified DC7 DA neurons which in wildtype controls express too little *th* to be detected by the technique. However, based on the fact that selective interference with Fgf3 signaling can compensate the formation of supernumerary DC7 DA neurons, we think that indeed additional DA neurons are observed in *cnot8^m1061^* mutant embryos.

Our findings reveal elevated *fgf3* mRNA levels in *cnot8^m1061^* mutant embryos, along with elevated levels of expression for the FGF responsive transcription factor genes *pea3* and *erm*. Given the expression domain of *fgf3* in the posterior wall of the caudal hypothalamus, we hypothesized that enhanced Fgf3 signaling may be the cause for formation of supernumerary DC7 DA neurons. To test this hypothesis, we blocked FGF signaling using the FGF receptor inhibitor SU5402. While DC7 DA neurons become postmitotic during an extended developmental period [44], we restricted our analysis to a shorter time window, because permanent global inhibition of FGF signaling affect pattern formation and embryonic survival. However, even when we restricted SU5402 application to 42 to 48 hpf only, we could reduce the wildtype and *cnot8^m1061^* mutant DC7 DA population by about half each. More specifically, when we eliminated Fgf3 activity in *lia* mutant embryos, we observed almost complete compensation of the supernumerary DC7 DA neurons in *cnot8^m1061^* mutant embryos. These data reveal that Fgf3 signaling contributes to specification of the number of DA neurons in the caudal hypothalamus. However, Fgf3 may not provide the only FGF activity, because residual DC7 DA neurons form in *lia*/*fgf3* homozygous mutant embryos. Other FGFs, including Fgf8a [63], may act redundantly with Fgf3. Fgf8 from the mid-hindbrain boundary has previously been linked to midbrain DA differentiation [73]. An alternative explanation would be that Fgf3 is not directly involved in controlling DC7 DA differentiation, but may rather control the size of the DC7 precursor pool size or proliferation of the precursor pool. A link for Fgf3 signaling to cell cycle control and differentiation in the hypothalamus has also been established for serotonergic neuron differentiation in zebrafish [63]. A regulation of DA neuron number by control of precursor pool size has also been shown for the more rostral posterior tubercular DA neurons in zebrafish, involving WNT signaling [45].

In summary, our data indicate that zebrafish Cnot8 contributes to regulation of proper transcript levels for a subset of developmental control and differentiation genes during embryogenesis, confirming conserved functions of Caf1, Pop2, and Cnot7/8 from yeast to vertebrates. The enhanced activity of Fgf3 signaling in the *cnot8* mutant helped us to uncover a role for Fgf3 in controlling the number of DA neurons developing in the zebrafish caudal hypothalamus. The *cnot8^m1061^* mutantion may be a useful model to further study the contribution and mechanisms of the Ccr4-Not complex towards control of transcript levels during development.

## Methods

### Ethics statement

This study was performed with the approval of the State of Baden-Württemberg Regierungspraesidium Freiburg Animal Protection Authorities in accordance with the German Animal Protection Act under permission number 35-9185.81/G-12/40.

### Fish maintenance, strains and genetics

Zebrafish were crossed and eggs maintained under standard conditions at 28,5°C [74]. Embryos were treated with 200 mM phenylthiourea to inhibit melanin pigmentation.

The *m1061* allele (Tübingen isolation number *t-03-0366*) was isolated in an ENU mutagenesis screen in a Tübingen wildtype strain genetic background [41]. Map crosses were set up between a heterozygous carrier of the *m1061* mutation and wild-type fish of the AB or HK strain.

The *lia(fgf3)^t24152^* mutant line was previously described [64]. Embryos were genotyped by PCR using the following primers: forward 5′ CAACCGAGAGTGTGAGTTTC 3′ and reverse 5′ CGTCCCTTTCCATTGATGGACAGATA 3′. PCR conditions: 94°C 2 min; 94°C 1 min; 56°C 1 min; 72°C 1 min; 35 cycles. The PCR product had a length of 200 bp. The *lia(fgf3)^t24152^* mutation generates an EcoRV site: PCR product digestion from yielded a 180 bp fragment indicative of the mutant allele.

### Mapping and cloning of *cnot8^m1061^*

We established a high resolution genetic mapping pannel consisting of 672 F2 embryos representing 1344 meioses from crosses of the original isolate *t-03-0366* into AB and HK strain polymorphic strains, providing approximately 0.07 cM resolution. Whole genome scan analysis using SSLP markers [46] linked the *m1061* mutation to chromosome 21. To yield additional polymorphic markers for fine mapping, genomic sequences in the critical region were obtained from Ensembl (Zv6, 7 and 8) genome browser and primer pairs for candidate SSLP markers were generated using the Zebrafish SSR search website, Massachusetts General Hospital, Charlestown USA. (http://danio.mgh.harvard.edu/markers/ssr.html). Fine mapping was performed using DNA from single embryos. BX294656.8 3p (forward, 5′-ATGTGCACCTGCAAAAGACA-3′; reverse, 5′-AATCAACCTCGTCATCCTCA-3′); CR855270.17 p9 (forward, 5′-TCATTATGCAGACTACATTTGAAAG-3′; reverse, 5-CCGTCCATTTGTTCATTCCT-3′); BX927237 p4 (forward, 5′-GAATGCAGGCGAATAGAACC-3′; reverse, 5-GCGAGACGCTCTAGGCTAGTT-3′); Zv8_scaffold2513.6 p2 (forward, 5′-TGTTGTTTGTGAGGAACTTAATGA-3′; reverse, 5-TCTCTCTTTTCTCAGCTGTGTTG-3′). Listed SSLP markers were also used to genotype *m1061* embryos. The full length *cnot8* ORF was amplified from cDNA of 3 dpf *cnot8^m1061^* mutant embryos and wild-type siblings (cnot8 p4 forward, 5′-GTTCCTCTGCCTTCATCATC-3′; reverse, 5′-AACTGCCTCGGTCAACAGAT-3′). PCR conditions: 94°C 2 min; 94°C 1 min; 60°C 1 min, 72°C 2 min, 30 cycles. Partial *cnot8* ORF was amplified from genomic DNA confirming the premature stop codon in *m1061* mutants (cnot8stop p1 forward, 5′-ATCCTTCAATAATCGCCATGT-3′; reverse, 5′-AACACAAAAGTAAGAAATGCTATTTGG-3′). PCR conditions: 94°C 2 min; 94°C 1 min; 60°C 1 min, 72°C 2 min, 30 cycles.

### Genotyping of *cnot8^m1061^* embryos

Following WISH, tail clips for DNA extraction were performed and the tightly linked BX294656.8 3p, CR855270.17 and p9 BX927237 p4 SSLP markers were used for genotyping comparing *cnot8^m1061^* mutant and wild-type embryos respectively.

### Plasmids and probes

*cnot8* digoxigenin-labeled sense probe was generated by linearizing the pCRIITOPO-*cnot8* plasmid with BamHI. *cnot8* digoxigenin-labeled antisense probe was generated linearizing the pCRIITOPO-*cnot8* plasmid with EcoRV. In addition, the following digoxigenin-labeled antisense probes were used: *th* [34], *fgf8* [51], *krox20/egr2b* [75], *emx1* [49], *nkx2.1a* [53], *otpa* [52], *sim1a, oxtl*, and *crh* [41], *tphd2* [61], *fgfr1-4* [76], *pea3* [77], *erm* [78], *fgf3* [79].

### Whole-mount *in situ* hybridization and fluorescent immunohistochemistry

Both methods were performed as previously described [80].

### Morpholinos

Morpholinos were obtained from Gene Tools LLC. Knock down of maternal and zygotic Cnot8 *was* achieved by coinjection of *1 ng of both MOcnot8ATG* (5′–ATGATGAAGGCAGAGGAACCAATTC–3′) and *p53ATG* Morpholino at 1 cell stage in wild-type embryos. *p53ATG* Morpholino was previously described [81].

### Cell counts and statistical analysis

Counts of *crh* and DA cells were performed visually using DIC transmitted light image stacks documenting the regions of the brain in which dopaminergic neurons are located. Cell counts were performed using ImageJ or Zeiss ZEN software. For all cell count analyses (Figures 1, 5, 9), cell numbers of 5 embryos for each experimental condition were determined. The numbers reported in the bar graphs are average numbers determined using the Microsoft Excel "Average" function, and plotted with standard deviation calculated by Microsoft Excel. Statistical analyses were performed using the nonparametric Mann-Whitney test by the statistical analysis software GraphPad Prism version 6.0d for MacOS, GraphPad Software, La Jolla California USA, www.graphpad.com.

### Image analysis

Images of WISH gene expression data were analyzed to determine whether significant differences in WISH signal intensities exist when wildtype control and mutant or experimental embryos were compared. To compare WISH signal intensities, control and experimental embryos were fixed and processed together. For mutant and WT embryos, both were processed together in one reaction tube, and genotypes determined by PCR after alkaline phosphatase stain reaction. Thus, WISH and stain procedures were under exactly identical conditions for samples to be compared. For each analysis, control and experimental WISH embryos were photographed in one session under identical imaging conditions (same orientation of embryo, comparable focal plane, same lens, DIC, and light setting on microscope, same exposure setting on ZEISS Axiocam MRc camera utilizing Axiovision software). For control and experimental embryos, typically five to six each were documented (see number of embryos N reported with experiments).

For quantification of stain intensities, images from experimental and control embryos to be compared were all assembled into one composite Photoshop (typically five controls on left side and five experimental embryos on right side) file, merged into one single layer separate from the background layer. Using the Photoshop "Levels" tool composite layers that did not fully use the 8-bit intensity range were adjusted linearly taking great care not to saturate pixels. The composite was flattened, and converted to grayscale (8 bit). The grayscale image was then inverted using the Image - Adjustments - Invert tool. These composite images were saved as TIFF files. TIFF files were opened using ImageJ 1.48o (64bit). For each individual image in the composite, the equivalent anatomical areas of WISH signal were marked using the freehand selection tool, drawn using a graph tablet (Bamboo Fun CTH-461; WACOM). Using the ImageJ Analyze - Measure command, the area as well as mean, minimum and maximum grey values were determined. Data were controlled such that maximum grey values did not exceed 254 (to exclude saturation). Data were transferred into Excel:mac2011.

For statistical analysis, data were transferred to GraphPad Prism version 6.0d for MacOS. Mann-Whitney tests (unpaired non-Gaussian data, nonparametric test) were performed to calculate two-tailed P values. P<0.05 was considered significant.

## Supporting Information

Figure S1 **Evaluation of Cnot8 ATG Morpholino knockdown phenotype.** Analysis of *fgfr1* expression at 1 dpf in wildtype embryos injected with cnot8 ATG Morpholino. (A, C) non-injected WT sibling. (B) injection of 1 ng cnot8 ATG Morpholino and 1 ng p53 Morpholino. (D) Injection of 2 ng cnot8 ATG Morpholino and 1 ng p53 Morpholino (A and B) lateral views.
(TIF)

Table S1 **Summary of statistical evaluations of cell count experiments and quantificational analysis of WISH signals for** **Figure 3** **–** **9** **.** The File contains the following sheets: Sheet 1 - Summary of results from image quantifications (green  =  significant differences using Mann-Whitney test; red  =  no significant differences). Sheet 2 - Results of Mann-Whitney tests for analysis of stain intensities in Figures 3 to 8. Sheet 3 - Image measurements Figure 3. Sheet 4 - Image measurements Figure 4. Sheet 5 - Image measurements Figure 5. Sheet 6 - Image measurements Figure 6. Sheet 7 - Image measurements Figure 7. Sheet 8 - Image measurements Figure 8. Sheet 9 - Summary of cell counts Figures 1 and 9. Sheet 10 - Statistical analysis of cell counts in Figure 1. Sheet 11 - Cell counts and statistical analysis Figure 5. Sheet 12 - Cell counts and statistical analysis Figure 9.
(XLSX)
