# Supplementary figures and images for: A Mutation in cnot8, Component of the Ccr4-Not Complex Regulating Transcript Stability, Affects Expression Levels of Developmental Regulators and Reveals a Role of Fgf3 in Development of Caudal Hypothalamic Dopaminergic Neurons
Source: PLoS One. 2014 Dec 5;9(12):e113829. doi: 10.1371/journal.pone.0113829 (PMC4257555; doi:10.1371/journal.pone.0113829)

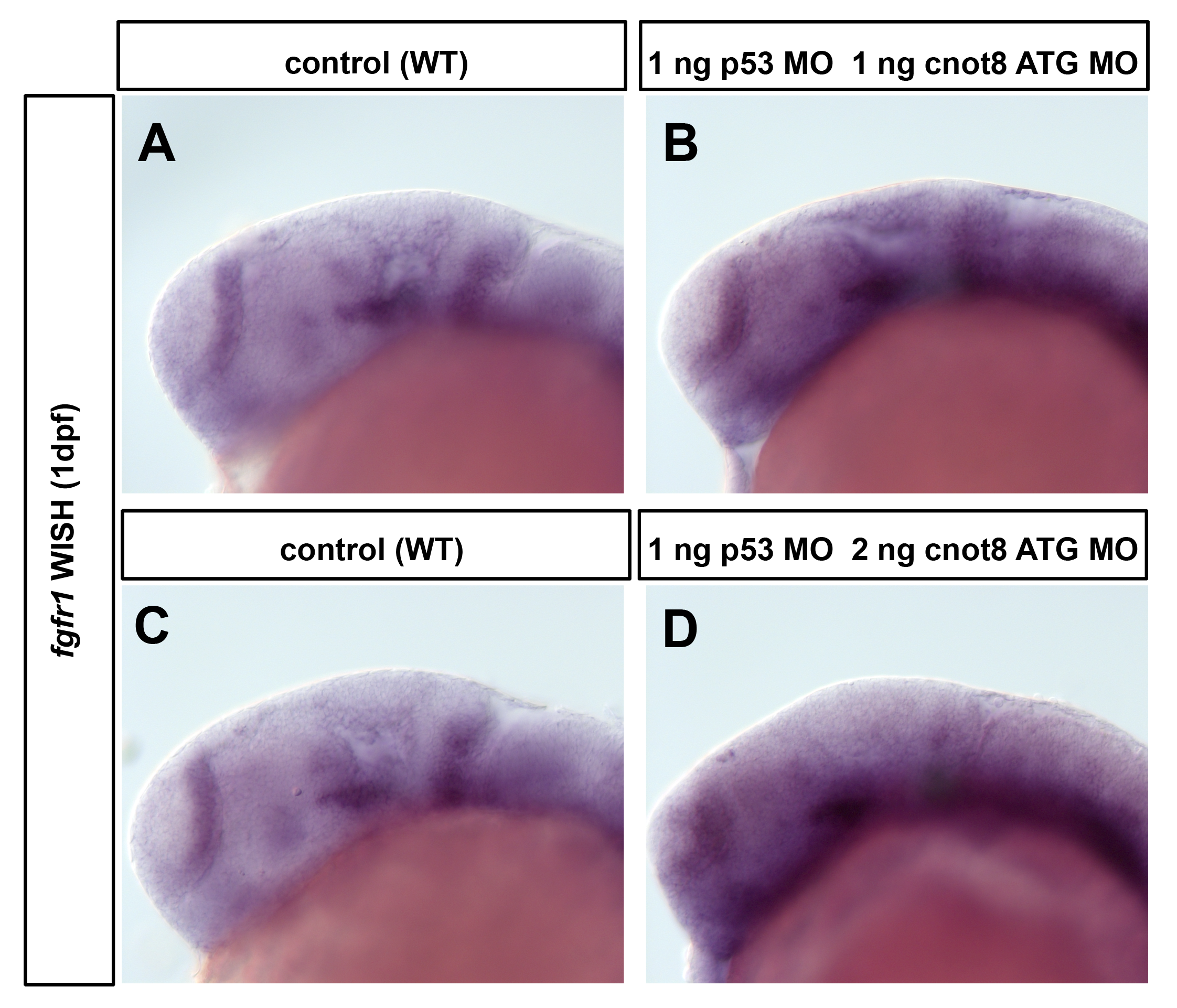

Supplement: Figure S1 — Evaluation of Cnot8 ATG Morpholino knockdown phenotype. Analysis of fgfr1 expression at 1 dpf in wildtype embryos injected with cnot8 ATG Morpholino. (A, C) non-injected WT sibling. (B) injection of 1 ng cnot8 ATG Morpholino and 1 ng p53 Morpholino. (D) Injection of 2 ng cnot8 ATG Morpholino and 1 ng p53 Morpholino (A and B) lateral views. (TIF) [file pone.0113829.s001.tif]
